# Metabolite profiling paradoxically reveals favorable levels of lipids, markers of oxidative stress and unsaturated fatty acids in a diabetes susceptible group of Middle Eastern immigrants

**DOI:** 10.1007/s00592-019-01464-w

**Published:** 2019-12-20

**Authors:** Mahmoud Al-Majdoub, Peter Spégel, Louise Bennet

**Affiliations:** 1 Unit of Molecular Metabolism, Department of Clinical Sciences in Malmö, Lund University Diabetes Centre, Lund University, Malmö, Sweden; 2 Centre for Analysis and Synthesis, Department of Chemistry, Lund University Diabetes Centre, Lund University, Lund, Sweden; 3 Department of Clinical Sciences, Family Medicine, Lund University, Skåne University Hospital, Building 28, Floor 11, Jan Waldenströms gata 35, 205 02 Malmö, Sweden; 4 Center for Primary Health Care Research, Region Skåne, Lund University, Malmö, Sweden

**Keywords:** Metabolomics, Migration, Middle East, Acylcarnitines, Fatty acids, Uremic solutes, Ketone bodies, Obesity, Type 2 diabetes, Hypertension

## Abstract

**Aims:**

The population of immigrants from the Middle East in Sweden show a higher prevalence of type 2 diabetes (T2D) compared to native Swedes. The exact reason for this is unknown. Here, we have performed metabolite profiling to investigate these differences.

**Methods:**

Metabolite profiling was conducted in Iraqi immigrants (*n* = 93) and native Swedes (*n* = 77) using two complementary mass spectrometry-based platforms. Differences in metabolite levels were compared after adjustment for confounding anthropometric, diet and clinical variables.

**Results:**

The Iraqi immigrant population were more obese (44.1 vs 24.7%, *p* < 0.05), but had a lower prevalence of hypertension (32.3 vs 54.8%, *p* < 0.01) than the native Swedish population. We detected 140 metabolites, 26 of which showed different levels between populations (*q* < 0.05,) after adjustment for age, sex, BMI, T2D and use of metformin. Twenty-two metabolites remained significant after further adjustment for HOMA-IR, HOMA-beta or insulin sensitivity index. Levels of polyunsaturated acylcarnitines (14:2 and 18:2) and fatty acid (18:2) were higher, whereas those of saturated and monounsaturated acylcarnitines (14:0, 18:1, and 8:1), fatty acids (12:0, 14:0, 16:0, and 18:1), uremic solutes (urate and quinate) and ketone bodies (beta-hydroxybutyrate) were lower in Iraqi immigrants. Further, levels of phospholipids were generally lower in the Iraqi immigrant population.

**Conclusions:**

Our result suggests an overall beneficial lipid profile in Iraqi immigrants, despite a higher risk to develop T2D. Higher levels of polyunsaturated fatty acids may suggest differences in dietary pattern, which in turn may reduce the risk of hypertension.

## Introduction

Studies have revealed an increased prevalence of metabolic diseases such as type 2 diabetes (T2D) in groups with low socioeconomic status [1], and certain ethnical minority and immigrant groups [2, 3]. One of the largest immigrant groups in Sweden is residents born in Iraq (Statistics Sweden). This group, and others from the Middle East, have an about twofold higher prevalence of T2D as compared to native Swedes [4, 5]. Parts of this difference could be attributable to higher prevalence of obesity, a family history of diabetes, differences in lifestyle, but also other unknown factors [6]. Differences in the prevalence of obesity between ethnic groups have shown to account for only a limited fraction of the increased T2D risk [7, 8]. Other studies have shown a higher frequency of T2D associated gene variants in some ethnic groups with high T2D risk [9]. However, diabetes susceptibility differs between geographical locations and the corresponding immigrant population, suggesting factors other than genetic predisposition to contribute to the increased risk [3]. Some of the remaining effects have instead been attributed to the impact of socioeconomic status [10], but also changes in dietary habits and physical activity [11].

In this study, we compare metabolite profiles in residents of Malmö, being born either in Sweden or in Iraq. Malmö is the largest city in southern Sweden, with approximately 300.000 inhabitants, out of which 32% being born abroad [12]. The ultimate aim is to find an explanation for the high prevalence of T2D in immigrants from the Middle East, thereby potentially allowing for tailored treatments that may reduce the risk of developing T2D in this population.

## Methods and procedures

### Study population

Between 2010 and 2012, a random sample of citizens of Malmö was selected from the census register and invited to participate in the MEDIM study (the impact of Migration and Ethnicity on Diabetes In Malmö). The recruitment process and exclusion criteria are described in detail elsewhere [13]. In total, 296 individuals were invited to participate, out of which 127 (43%) were born in Iraq and the rest in Sweden. *Finally, 96 men and women born in Iraq and 79 born in Sweden participated in the study.* In this study, we included 93 Iraqis and 77 Swedes, for whom complete data were available. All participants gave written informed consent. The study was approved by the ethics committee at Lund University (No. 2009/36 and 2010/561) and conforms to the principles outlined in the Declaration of Helsinki.

### Physical examination, blood analyses and questionnaires

Anthropometric data and data on clinical variables were collected by specially trained nurses. The procedures for the investigations are described in detail elsewhere [13]. Briefly, diagnostic blood tests were done after a 10-h fast. Information on lifestyle (including dietary data), family history of disease, comorbidity, pharmacological treatment, education level, etc., was collected by Arabic and Swedish speaking nurses. Dietary data contained information on the frequency of intake of vegetables, fruits and berries, seafood, soft drinks and sweets and were based on diet indices by the Swedish National Board of Health and Welfare [14]. These indices reflecting healthy eating habits are based on nutrition recommendations outlined by the Swedish Food Administration [15]. Assessments of insulin secretion (HOMA-beta) and resistance (HOMA-IR; Insulin sensitivity index (ISI)) were conducted as described previously [13].

### Metabolite profiling

Metabolites were profiled using two complementary platforms. Low-molecular weight polar to non-polar metabolites were analyzed by gas chromatography/time-of-flight mass spectrometry (GC/TOF-MS), as previously described in detail [16]. Lipophilic metabolites were analyzed by reversed-phase ultrahigh performance liquid chromatography/quadrupole-TOF-MS (UHPLC/QTOF-MS) [17]. Samples were analyzed in a randomized order with a quality control sample, produced by pooling a small aliquot from all samples, analyzed after every tenth sample injection. Metabolites were identified using the NIST, Golm and an in-house library (GC/TOF-MS) and Metlin MS/MS library (UHPLC/QTOF-MS) and confirmed by injection of pure standards, when available.

### Statistical analysis

Statistical analyses were performed in R version 3.3.3. Categorical clinical, anthropometric and lifestyle variables were compared using a Chi-squared test, continuous normally distributed data using the Student’s *t* test and non-normally distributed data using the Wilcoxon signed-rank test (stats package). Metabolites were log2-transformed, and linear regression models were built using lmFit (limma package), using an empirical Bayes approach to evaluate differences (ebayes, limma package). Significance was defined as *q* < 0.05 using multiple testing correction according to the false discovery rate method (p. adjust, stats package).

### Ethical considerations

All participants have provided written informed consent to participate. The Ethics Committee at Lund University approved the study (application nos. 2009/36 and 2010/561). This investigation conforms to the principles outlined in the Declaration of Helsinki [18].

## Results

### Clinical characteristics

Anthropometric, clinical and lifestyle factors for the study population have been summarized previously [12]. Here, we just summarize some key variables in the subset that was examined in this report (Table 1). BMI was slightly higher in the Iraqi participants, with 44.1% being defined as obese (BMI ≥ 30 kg/m^2^), as compared to 24.7% among Swedish participants (*p* < 0.05). However, the waist-to-hip-ratio did not differ between the groups. Swedish participants were on average 3 years older (*p* < 0.001), had a 26% higher prevalence of hypertension (*p* < 0.01), slightly higher plasma alanine amino transferase (ALAT; *p* < 0.05) and had more often a university degree (*p* < 0.001). Iraqi born participants had higher levels of triglycerides (*p* < 0.01) and lower HDL (*p* < 0.01). Notably, beta-cell function, as estimated by HOMA-beta, was enhanced in the Iraqi participants (*p* < 0.01). Differences in BMI, obesity, education, HDL, systolic blood pressure, hypertension and ASAT were independent of age and sex, with the latter variables also independent of BMI (*p* < 0.05). Regarding lifestyle, physical inactivity was prevalent in both Iraqi and Swedish born participants (63% vs 60%, respectively, *p* = 0.26) and there were no differences in smoking habits. Regarding dietary data, we observed very few differences between the groups, with only consumption of soft drinks being higher among the Iraqis (*p* = 0.004). All metabolites, except for fumarate, remained significant after adjustment of models for these parameters. Seven Iraqi reported that they consumed alcohol and 7 that they did not consume alcohol, with 79 not responding. For Swedes, the corresponding numbers were 47, 20 and 10.

**Table 1:** Characteristics of the sample of MEDIM study participants from Sweden and Iraq selected for metabolite profiling

Variables	Iraqi (*n* = 93) (median/*n*; (%)/[IQR])	Swedes (*n* = 77) (median/*n*; (%)/[IQR])	*p*
Sex (female) (*n*)	50 (53.8)	42 (54.5)	ns
Age (years)	54.1 [48.6–58.7]	57.7 [52.4–60.3]	0.006
BMI (kg/m^2^)	29.3 [26.3–31.89]	26.2 [23.8–29.7]	0.0007
Waist/hip-ratio	0.92 [0.87–0.97]	0.93 [0.76–0.97]	ns
Obesity, BMI ≥ 30 kg/m^2^ (*n*)	41 (44.1)	19 (24.7)	0.013
Fasting glucose (mmol/L)	5.6 [5–6.4]	5.8 [5.3–6.4]	ns
HbA1c (mmol/mol)	40.6 [37.5–43.7]	39.5 [38.5–42.7]	ns
ISI	84.4 [64.6–120.1]	104.2 [69.5–147.5]	ns
HOMA-IR	2 [1.3–3.1]	1.9 [1.2–2.5]	ns
HOMA-beta	74.9 [42.6–114.9]	60 [42.11–88.3]	0.0091
IGT (*n*)	14 (15.1)	7 (9.1)	ns
T2D (*n*)	19 (20.4)	14 (18.2)	ns
Metformin (*n*)	15 (16.1)	5 (6.5)	ns
Systolic blood pressure (mmHg)	1.28 [1.18–1.44]	1.42 [1.3–1.5]	0.00095
Diastolic blood pressure (mmHg)	81.5 [74–87.5]	86.5 [80–93.5]	0.027
Hypertension (*n*)	30 (32.3)	45 (58.4)	0.0011
Cholesterol (mmol/L)	5.1 [4.4–5.8]	5.4 [4.6–6.2]	ns
TG (mmol/L)	1.4 [1–2.1]	1 [0.7–1.6]	0.0022
HDL (mmol/L)	1.01 [0.83–1.2]	1.18 [0.92–1.5]	0.0031
LDL (mmol/L)	3.3 [2.8–4]	3.5 [2.8–4.1]	ns
LDL/HDL	3.4 [2.7–4.1]	3.1 [2.2–4.1]	ns
ASAT (µkat/L)	0.33 [0.29–0.4]	0.38 [0.32–0.46]	0.013
ALAT (µkat/L)	0.37 [0.29–0.54]	0.4 [0.29–0.59]	ns
Soft drinks, more than once a week	51	31	0.004
Vegetables, less than twice a week	7	10	ns
Fruit/berries, less than twice weekly	10	11	ns
Fish, less than once a week	32	23	ns
Sweets, more than twice a week	49	51	ns
Smoking (*n*)	28 (30.1)	29 (37.7)	ns
Physically inactive (*n*)	59 (63)	46 (60)	ns
University education (*n*)	41 (44.1)	59 (76.6)	0.000028

*ISI* insulin sensitivity index, *CIR* corrected insulin response, *DI* disposition index, *IGT* impaired glucose tolerance, *T2D* type 2 diabetes, *TG* triglyceride, *ASAT* aspartate amino transferase, *ALAT* alanine amino transferase

### Metabolite profiles

In total, 140 metabolites were relatively quantified by our two complementary metabolite profiling platforms. These included 22 amino acids, 8 fatty acids, 19 acylcarnitines and 41 phospholipids. First, we examined differences in metabolite levels between the Iraqi and Swedish participants, after adjustment for age, sex, BMI, diabetes diagnosis and use of metformin. In total, 26 metabolites showed different levels between the groups (Table 2). These included higher levels of carnitines 14:2, 18:2, and 3:0 and linoleate (18:2) in Iraqi participants. On the contrary, levels of carnitine 14:0, 18:1 and 8:1, and fatty acids 12:0, 14:0, 16:0 and 18:1, as well as the ketone body beta-hydroxybutyrate, the oxidative stress marker alpha-hydroxybutyrate and the uremic retention solutes urate and quinate were lower in Iraqi participants, as compared to native Swedes. Differences remained after further adjustment for HOMA-beta, HOMA-IR, ISI and HOMA-IR + diet (Table 3), except for carnitine 10:1, PC 34:1, linoleate, ribitol and fumarate which were no longer significant.

**Table 2:** Metabolites differing between groups, after adjustment for sex, age, BMI, diabetes diagnosis and metformin use

Metabolite	log(FC)^a^	*p*	*q*
Quinate	− 1.77 [− 2.04, − 1.51]	4.00E−13	5.60E−11
C 12:0	− 1.2 [− 1.46, − 0.94]	3.88E−11	2.71E−09
C 14:0	− 0.58 [− 0.97, − 0.19]	1.19E−06	4.35E−05
Carn 14:0	− 0.52 [− 0.64, − 0.4]	1.29E−06	4.35E−05
β-Hydroxybutyrate	− 0.71 [− 0.91, − 0.51]	1.55E−06	4.35E−05
2-Hydroxyglutarate	− 0.43 [− 0.7, − 0.17]	4.12E−06	9.62E−05
Carn 14:2	0.67 [0.48,0.87]	5.23E−06	0.0001
Carn 18:2	0.52 [0.4,0.65]	1.77E−05	0.00029
LPC 20:5	− 0.78 [− 1.44, − 0.12]	1.88E−05	0.00029
PC 32:1	− 0.55 [− 0.71, − 0.39]	4.28E−05	0.0006
LPE 20:5	− 0.55 [− 1.07, − 0.02]	7.12E−05	0.00091
LPC 16:1	− 0.41 [− 0.86,0.03]	0.00011	0.00127
Carn 3:0	0.41 [0.13,0.69]	0.00033	0.00357
SM 32:1	− 0.51 [− 0.66, − 0.37]	0.00048	0.00446
C 16:0	− 0.33 [− 0.53, − 0.12]	0.00048	0.00446
Carn 8:1	− 0.6 [− 0.74, − 0.46]	0.00093	0.00817
Carn 10:1	0.46 [0.3,0.61]	0.0018	0.01485
C 18:1	− 0.32 [− 0.68,0.03]	0.00262	0.02034
2-Aminobutyrate	− 0.24 [− 0.55,0.07]	0.00332	0.02445
Carn 18:1	− 0.34 [− 0.46, − 0.22]	0.00569	0.03986
C 18:2	0.28 [0.02,0.55]	0.00608	0.04052
Urate	− 0.27 [− 0.54,0]	0.00753	0.04597
α-Hydroxybutyrate	− 0.25 [− 0.48, − 0.02]	0.00762	0.04597
Malate	− 0.26 [− 0.54,0.03]	0.00788	0.04597
Ribitol	− 0.23 [− 0.5,0.04]	0.00857	0.04627
PC 34:1	− 0.24 [− 0.46, − 0.02]	0.00859	0.04627

*Carn* carnitines, *C* fatty acid, *PC* phosphatidylcholine, *SM* sphingomyelin, *LPC* lysophosphatidylcholine, *LPE* lysophosphatidylethanolamine, followed by carbons:unsaturations

^a^*FC* fold-change; shown as log(fold-change) [left, right limit of confidence interval]

**Table 3:** Metabolites differing between groups, after adjustment for sex, age, BMI, diabetes diagnosis, metformin use (model) and HOMA-beta, HOMA-IR, ISI or HOMA-IR + diet

	Model + HOMA-B		Model + HOMA-IR		Model + ISI		Model + HOMA-IR + Diet	
	FC	*q*	FC	*q*	FC	*q*	FC	*q*
Quinate	− 1.82	4.2E−10	− 1.83	2.47E−10	− 1.8	7.4E−10	− 1.75	2.78E−09
C 12:0	− 1.28	1.26E−08	− 1.29	9.46E−09	− 1.29	1.12E−08	− 1.24	1.89E−08
b-hydroxybutyrate	− 0.78	0.0000709	− 0.77	0.0000889	− 0.77	0.0000959	− 0.74	0.000171
C 14:0	− 0.63	0.00012	− 0.63	0.00013	− 0.64	0.0000959	− 0.55	0.000495
Carn 14:0	− 0.55	0.00012	− 0.55	0.00013	− 0.55	0.00013	− 0.59	3.09E−05
LPC 20:5	− 0.81	0.00155	− 0.8	0.00164	− 0.79	0.00209	− 0.95	8.65E−05
Carn 14:2	0.62	0.00208	0.62	0.00246	0.62	0.00235	0.68	0.000495
PC 32:1	− 0.54	0.00208	− 0.54	0.00246	− 0.55	0.00235	− 0.62	0.000575
2-Hydroxyglutarate	− 0.39	0.00208	− 0.39	0.00242	− 0.39	0.00235	− 0.50	7.97E−05
LPE 20:5	− 0.56	0.00222	− 0.56	0.00246	− 0.56	0.00268	− 0.61	0.000815
Carn 3:0	0.45	0.00281	0.45	0.00393	0.44	0.00483	0.37	0.0176
LPC 16:1	− 0.41	0.00337	− 0.41	0.00393	− 0.4	0.00483	− 0.55	8.65E−05
Carn 18:2	0.47	0.00396	0.47	0.00415	0.47	0.00445	0.48	0.00269
C 16:0	− 0.34	0.01046	− 0.34	0.01129	− 0.35	0.00745	− 0.36	0.00531
Malate	− 0.29	0.01813	− 0.29	0.01999	− 0.3	0.0151	− 0.32	0.00729
SM 32:1	− 0.47	0.01897	− 0.48	0.0222	− 0.49	0.01972	− 0.64	0.000673
C 18:1	− 0.35	0.02891	− 0.35	0.0314	− 0.36	0.0245	− 0.33	0.0293
2-Aminobutyrate	− 0.24	0.02891	− 0.24	0.0314	− 0.24	0.03261	− 0.29	0.00716
Carn 18:1	− 0.37	0.04764	− 0.37	0.04817	− 0.38	0.03852	− 0.44	0.00825
Carn 8:1	− 0.53	0.04814	− 0.53	0.04881	− 0.55	0.03443	− 0.72	0.00238
Fumarate					− 0.25	0.04129	ns	
Urate					− 0.25	0.03852	− 0.28	0.0176

*Carn* carnitines, *C* fatty acid, *PC* phosphatidylcholine, *SM* sphingomyelin, *LPC* lysophosphatidylcholine, *LPE* lysophosphatidylethanolamine followed by carbons:unsaturations

## Discussion

Several studies indicate that ethnic minorities in European and Nordic countries have a higher prevalence of diabetes, which has been attributed to differences in genetic predisposition, socioeconomic factors, physical activity and diet [2, 3]. However, independent of the cause of this observation, the huge costs and suffering associated with T2D in these groups need to be acted upon. In the present study, we have performed metabolite profiling in immigrants born in Iraq and compared result with individuals born in Sweden, aiming at producing a detailed phenotyping of these two groups.

Both populations showed a prevalence of obesity, defined as BMI ≥ 30 kg/m^2^, which was higher than the 19% estimated by the Public Health Agency of Sweden from BMI for the population of > 45 years of age. Notably, obesity was more prevalent, and BMI higher, among Iraqi immigrants, whereas there was no difference in the waist-to-hip-ratio. Hence, fat distribution is likely to differ between Iraqi and Swedes.

In total, we found 26 metabolites to show different levels between Iraqis and Swedes, independent of sex, age, BMI, diabetes diagnosis and metformin use. Out of these, 22 remained significant after further adjustment for measures of insulin resistance and beta-cell function. Notably, levels of short saturated (12:0, 14:0 and 16:0) and monounsaturated (18:1) fatty acid were lower in Iraqi, whereas the polyunsaturated fatty acid (18:2) was higher. These differences were also reflected in levels of acylcarnitines; saturated acylcarnitine (14:0) which showed lower levels and polyunsaturated (14:2 and 18:2) higher levels in Iraqi immigrants. Moreover, HOMA-beta was higher in the Iraqi population, which is in line with a vast number of in vitro studies, suggesting unsaturated fatty acids to improve beta-cell function [19]. Prolonged fasting has been shown to increase levels of polyunsaturated fatty acids and unsaturated acylcarnitines more than those of the saturated intermediates [20]. One might hypothesize that a possible explanation for the difference between groups with respect to acyl unsaturation would be related to different durations of fasting prior to sampling. However, all participants fasted for an equal long time and the hypothesis is further contradicted by the observed higher levels of the ketone body beta-hydroxybutyrate in the Swedish group, levels of which increase during starvation [20].

Despite a higher prevalence of obesity in the Iraqi immigrant population, prevalence of hypertension was surprisingly lower than in Swedes. This was independent of age, sex and BMI. Whether this is due to differences in fat distribution or any other factor remains to be determined. Hypertension is often associated with reduced kidney function [21]. In line with this, the uremic retention solutes urate and quinate [22] showed higher levels in Swedish participants. The prevalence of hypertension has previously been shown to be lower in non-European immigrants living in Sweden, the majority of which are from the Middle East, as compared to native Swedes [23]. In line with this, the prevalence of hypertension was found to be similar in Americans originating from Europe and the Middle East, whereas T2D was more prevalent in the latter population [24]. Further, we have previously shown, in the larger MEDIM cohort including over 2000 individuals, a more favorable kidney function in the Iraqi immigrant than native Swedish population [25]. Whether these observations relate to the lower blood pressure and more favorable lipid profile in the Iraqi population remain to be further investigated.

Our observation of higher levels of polyunsaturated fats in the Iraqi population, but also the presumably lower alcohol consumption and lower blood pressure levels [23] may suggest a dietary origin for the observed differences in hypertension between the populations [26]. We could not observe any differences in dietary habits across Middle Eastern and European ethnicities, but for soda consumption. Data on food habits were gathered using food questionnaires recommended by the Swedish National board of Welfare capturing dietary indices as indicators of unhealthy eating habits. However, these questionnaires collecting food frequency but neither portion size nor food preparation may not fully reflect the content of eating habits, including calories, fat and sugar intake. Since approximately 70% of Iraqi immigrants eat traditional Iraqi food [27] which is commonly deep-fried, a large proportion of the fat intake in this population may come from fat sources such as olive oil, which are rich in unsaturated fats. In a previously conducted lifestyle intervention study addressing Iraqi immigrants at high T2D risk, we collected 4-day food diaries assessing frequency of intake, but also portion size and food preparation. In that study, dietary fat intake was twice as high in the Iraqi immigrant population as compared to the native Swedish population, with over 40% of total energy intake coming from fat [28]. The high fat content may impact the outcome of our data, and we conclude that although the questionnaires used in this study are validated, they may not fully reflect food composition and eating habits. A detailed and long-term investigation of dietary patterns in this population is currently missing.

All phospholipids detected on our platforms showed higher levels in Swedish participants. Most of these lipids belonged to the phosphatidylcholine and phosphatidylethanolamine classes, which are enriched in HDL particles [29]. In line with this, HDL showed higher levels in Swedish, as compared to Iraqi, participants.

Interestingly, levels of alpha-hydroxybutyrate, a marker of oxidative stress and an early marker of insulin resistance [30], also showed higher levels in Swedes, despite of similar HOMA-IR in the two populations.

A strength of this study is the study design from the census register randomly inviting residents born in Iraq or Sweden living in the same socioeconomic neighborhood and the thorough sampling of phenotype besides anthropometrics including lifestyle, pharmacological treatment and metabolism [13]. The participation rate was almost 80% in Iraqi born immigrants and over 45% in native Swedes, thus reflecting a representative study sample. A limitation of the study is the cross-sectional design, precluding any causal inference. Another potential limitation is the relatively small study sample which might have influenced our results. However, a larger study was conducted a few years later including over 2000 participants born in Iraq or Sweden [6]. Since anthropometrics and biochemistry do not differ between these studies and we have shown that non-participants have similar comorbidity in terms of rates of T2D, we consider our data reliable.

In conclusion, metabolite profiling in Iraqi immigrants, a population with an increased risk of develop diabetes [4, 5], despite adjusting for diet revealed higher levels of metabolites previously indicated to be health promoting, including unsaturated fatty acids and their metabolic products. Further investigations are needed to establish the physiological role of the observed metabolic differences.

## Abbreviations

ALAT: Alanine amino transferase
ASAT: Aspartate amino transferase
CIR: Corrected insulin response
DI: Disposition index
IGT: Impaired glucose tolerance
ISI: Insulin sensitivity index
MS: Mass spectrometry
T2D: Type 2 diabetes
TG: Triglyceride
UHPLC: Ultrahigh performance liquid chromatography
